# Vascular adhesion protein-1 is actively involved in the development of inflammatory lesions in rat models of multiple sclerosis

**DOI:** 10.1186/s12974-018-1152-2

**Published:** 2018-05-01

**Authors:** Petri Elo, Sina Tadayon, Heidi Liljenbäck, Jarmo Teuho, Meeri Käkelä, Kalle Koskensalo, Virva Saunavaara, Jenni Virta, Tibor Z. Veres, Aida Kiviniemi, Antti Saraste, Päivi Marjamäki, Laura Airas, Sirpa Jalkanen, Anne Roivainen

**Affiliations:** 1 Turku PET Centre, University of Turku, Kiinamyllynkatu 4-8, FI-20520 Turku, Finland; 2 Turku Center for Disease Modeling, University of Turku, Kiinamyllynkatu 10, FI-20520 Turku, Finland; 3 Turku PET Centre, Turku University Hospital, Kiinamyllynkatu 4-8, FI-20520 Turku, Finland; 4 Department of Medical physics, Turku University Hospital, Kiinamyllynkatu 4-8, FI-20520 Turku, Finland; 5 MediCity Research Laboratory, University of Turku, Tykistökatu 6, FI-20520 Turku, Finland; 6 Department of Radiology, Medical Imaging Centre of Southwest Finland, Turku University Hospital, Kiinamyllynkatu 4-8, FI-20520 Turku, Finland; 7 Division of Clinical Neurosciences, Turku University Hospital, Kiinamyllynkatu 4-8, FI-20520 Turku, Finland

**Keywords:** Adhesion molecule, Experimental allergic encephalomyelitis, Blood brain-barrier, Inflammation, Multiple sclerosis

## Abstract

**Background:**

Vascular adhesion protein-1 (VAP-1) is an inflammation-inducible endothelial cell molecule and primary amine oxidase that mediates leukocyte entry to sites of inflammation. However, there is limited knowledge of the inflammation-related expression of VAP-1 in the central nervous system (CNS). Therefore, we investigated the expression of VAP-1 within the CNS vasculature in two focal rat models of experimental autoimmune encephalomyelitis (EAE) mimicking multiple sclerosis (MS).

**Methods:**

EAE was induced either with Bacillus Calmette-Guérin, resulting in a delayed-type hypersensitivity-like pathogenesis (*f*DTH-EAE), or with myelin oligodendrocyte glycoprotein (*f*MOG-EAE). A subgroup of *f*MOG-EAE rats were treated daily with a selective VAP-1 inhibitor (LJP1586; 5 mg/kg). On 3 and 14 days after lesion activation, rat brains were assessed using magnetic resonance imaging (MRI), and ex vivo autoradiography was conducted to evaluate the binding of Gallium-68-labelled VAP-1 ligand. Histology and immunohistochemistry (OX-42, VAP-1, intercellular adhesion protein-1 [ICAM-1], P-selectin) supported the ex vivo autoradiography*.*

**Results:**

EAE lesions showed MRI-detectable signal changes and binding of the VAP-1-targeting radiotracer in both rat models. Some of the VAP-1 positive vessels showed morphological features typical for high endothelial-like venules at sites of inflammation. Inhibition of VAP-1 activity with small molecule inhibitor, LJP1586, decreased lymphocyte density in the acute inflammatory phase of *f*MOG-EAE lesions (day 3, *P* = 0.026 vs. untreated), but not in the remission phase (day 14, *P* = 0.70 vs. untreated), and had no effect on the amount of OX-42-positive cells in either phase. LJP1586 treatment reduced VAP-1 and ICAM-1 expression in the acute inflammatory phase, whereas P-selectin remained not detectable at all studied stages of the disease.

**Conclusions:**

Our results revealed that VAP-1 is expressed and functionally active in vasculature within the induced focal EAE lesions during the acute phase of inflammation and remains expressed after the acute inflammation has subsided. The study indicates that VAP-1 is actively involved in the development of inflammatory CNS lesions. During this process, the endothelial cell lesion-related vasculature seem to undergo a structural transformation from regular flat-walled endothelium to HEV-like endothelium.

## Background

Multiple sclerosis (MS) is a common chronic disease that causes progressive disability and often affects young adults. MS is highly variable with respect to symptoms, disease progression and pathogenesis. It is, therefore, categorized into clinically different subtypes. Each subtype involves an inflammatory component that has an impact on symptoms and disease progression [1]. Although the pathophysiology of the progression of MS still remains relatively unexplored, the extent of microglial activation and the influx of inflammatory cells into the central nervous system (CNS) are considered fundamental in MS pathogenesis [2, 3]. The infiltration of inflammatory cells into the tissue requires interaction between leukocyte ligands and their counter-receptors on the endothelial cells. The adhesive properties of a vessel wall are regulated by several adhesion molecules, such as selectins, intercellular adhesion molecule-1 (ICAM-1), vascular cell adhesion molecule-1 and vascular adhesion protein-1 (VAP-1) [4].

VAP-1 is a 170-kD endothelial cell surface molecule that controls the trafficking of immune cells to the sites of inflammation [5]. Upon inflammation, VAP-1 is upregulated in the endothelial cells and translocated from the intracellular storage granules to the luminal side of vessels. This is a key element in the control of the leukocyte extravasation during inflammation [5]. Relatively prominent expression levels of VAP-1 are found in high endothelial venules (HEV) in secondary lymphoid tissues [6–8]. VAP-1, therefore, plays a crucial role in regulating immune response in many inflammatory conditions. Translocation of VAP-1 to cell surface might also occur in MS as VAP-1 is suspected to actively transport the recruited leukocytes to the sites of inflammatory lesions. Previous findings from patients with MS show occasional VAP-1 positivity in brain microvessels, but the majority of the blood vessels within the brain parenchyma appear to lack VAP-1 [9]. This suggests that VAP-1 is stored at low basal concentrations in brain vessels to enable the rapid amplification of immune responses against pathogens in CNS. In addition to the membrane-bound VAP-1, a soluble form of VAP-1 (sVAP-1) is present in serum, and according to its amino acid sequence, sVAP-1 is believed to be derived from the membrane-bound form of VAP-1 [10]. The soluble form is thought to facilitate leukocyte binding to endothelial cells through interaction with its leukocyte counter-receptor, thus enhancing the adhesive status of leukocytes. Increased sVAP-1 expression has been observed in many different chronic inflammatory conditions, including inflammatory liver disease, skin inflammation, synovitis and active relapsing-remitting MS (RR-MS) [2, 10–12]. However, information is limited on the functional role of VAP-1 in CNS inflammation. Treatment with VAP-1 inhibitors has proven beneficial in experimental autoimmune encephalomyelitis (EAE), indicating that VAP-1 might play a role in inflammatory lesions within CNS [11].

Leukocyte transmigration is facilitated by blood-brain barrier (BBB) breakdown, which provides a mechanism for leukocytes to access CNS parenchyma during inflammation [13]. Changes in BBB status during neuroinflammation are categorized into BBB disruption and BBB activation. Disruption refers to leakage of the vessel wall and the loss of tight junctions, whereas BBB activation translates to the increased expression of adhesion molecules, such as VAP-1, by endothelial cells and astrocytes that amplify the immune response against CNS lesions [13]. VAP-1 function in relation to relapses is not well characterized in MS or EAE. During relapses in active RR-MS, there is BBB breakdown as demonstrated by Gadolinium (Gd)-enhancement of the active lesions seen in magnetic resonance imaging (MRI) [14]. It is reasonable to suspect that the lesion-related BBB remains abnormal also during remissions. This might affect the disease progression between relapses, the relapse rates and the response to anti-adhesive therapy. In addition, the characterization of early lesion development is a current trend with growing importance in MS research. Evidence suggests that the pathological stage and cellular components of pre-active MS lesions are distinct from active MS lesions, which typically present demyelinating axons, BBB breakdown and perivascular leukocyte infiltration [15]. As microglial activation is believed to be crucial in driving the pre-active MS lesion formation [15, 16], the expression of activated microglia, in this study, was used as a positive control for EAE lesion characterization and for confirming novel findings in early lesion development.

MRI is typically used as a standard tool for diagnosing MS because the T2-weighted and Gd-enhanced T1-weighted sequences can detect white matter demyelinating lesions, edema and inflammation throughout the brain and spinal cord [17]. MRI can also be used to monitor the lesion size and the extent of MS plaques and to ascertain BBB integrity [18, 19]. A clinical 3-T MRI with a dedicated small animal brain coil has shown potential for imaging rat brain glioma [20], but has not yet been applied to the evaluation of focal models of EAE in rat.

In this study, we investigated whether VAP-1 is expressed in inflammatory CNS lesions, whether it is involved in the development of inflammatory CNS lesions in rat models of MS, and whether VAP-1 is expressed on blood vessels in areas of normal appearing white matter (NAWM). In addition, we tested a new 3-T MRI protocol especially designed to visualize MS-like lesions in rat brain, which can be used to acquire supportive data regarding the lesion size and BBB status when using Gd-based contrast agents. Ex vivo autoradiography with a VAP-1 targeted positron emission tomography (PET) tracer, i.e. Gallium-68-labelled 1,4,7,10-tetraazacyclododecane-*N,N′,N′′,N′′′*-tetraacetic acid conjugated sialic acid-binding immunoglobulin-like lectin 9 motif containing peptide (^68^Ga-DOTA-Siglec-9), and immunohistochemistry were used to establish the spatial and temporal relationship between VAP-1 expression, hallmarks of leukocyte recruitment and lesion evolution.

## Methods

### Animals and study design

To gain insight into the inflammation and VAP-1 expression patterns in different disease subtypes, two different animal models mimicking MS were used. The focal delayed-type hypersensitivity (*f*DTH)-induced EAE is a representative of type I immunopathology of MS, and the focal myelin oligodendrocyte glycoprotein (*f*MOG)-induced EAE is a representative of type II immunopathology [18]. Both models had two subgroups that were studied on days 2−3 and days 13−14 post lesion activation. In addition, a subset of *f*MOG-EAE rats (*n* = 8) were administered daily with a selective VAP-1 inhibitor LJP1586 (Z-3-fluoro-2-(4-methoxybenzyl) allylamine hydrochloride, (SYNthesis med chem**,** Monash Institute of Pharmaceutical Sciences, Melbourne, Australia), 5 mg/kg intraperitoneally (i.p) for 3 or 14 days starting from the activation of the disease [21]. In addition to EAE models, sham-operated rats were studied as controls.

A total of 32 male Lewis rats (227 ± 6.0 g) were acquired from Charles River, Germany. The rats were allowed to familiarize themselves with the animal house environment for 5 days before starting any experiments. Dried pellet food and tap water were available *ad libitum*. Daily 12-h light and 12-h dark rhythm was maintained, and room temperature was kept at 21 °C during the whole study.

**Fig. 1:**
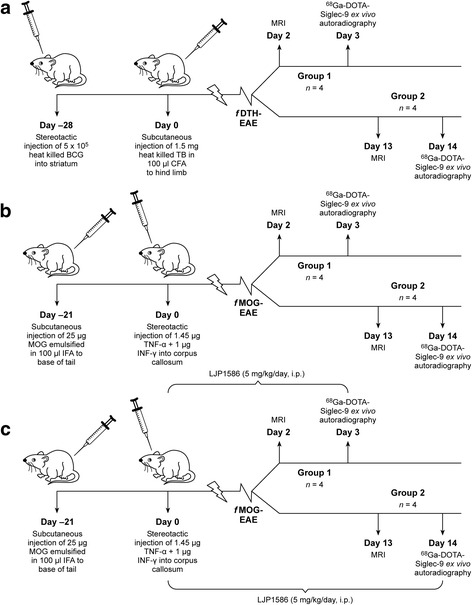
Protocols for the type I (**a**) *f*DTH-EAE, type II (**b**) *f*MOG-EAE and (**c**) LJP1586 treated *f*MOG-EAE experimental models

The study protocol is presented in Fig. 1. First, the rats were imaged using 3-T MRI to evaluate the lesion size and the status of BBB integrity using a Gd-based contrast agent. Then, ex vivo autoradiography studies were conducted to assess the binding of VAP-1 targeting ^68^Ga-DOTA-Siglec-9 in the vasculature of brain lesions. In addition, expression of VAP-1 was determined by immunofluorescence. ICAM-1 and P-selectin-detecting immunofluorescence and microglia-detecting immunohistochemistry were performed to support the findings*.* For immunohistochemistry, the marker of microglial activation (OX-42) was considered as a positive sign of EAE lesion development.

### *f*DTH-EAE, type I animal model of MS

Eight rats were first anesthetized with a mixture of 4–5% isoflurane and oxygen (500–700 mL/min), and then, the rats were administered with a subcutaneous (s.c.) injection of 100-μL Temgesic (0.05 mg/kg). After induction, anesthesia was maintained with 2–2.5% isoflurane (400–500 mL/min) and the rats were allowed to lie on a heating pad to sustain the body temperature. The rats were set on a stereotactic frame in order to fix the head in a coordinated system. A short incision was made to the scalp for the purpose of drilling a 1.0-mm hole at 1.0 mm anterior and 3.0 mm lateral from the bregma. Suspension (5 × 10^5^ organisms in 1 μL of saline) of 2 μL of heat-killed Bacillus Calmette-Guérin (BCG; a kind gift from Professor Daniel Anthony, Department of Pharmacology, University of Oxford, UK) was injected using a Hamilton micro-syringe (Hamilton Bonaduz AG, Bonaduz, Switzerland) at a depth of 4.0−2.5 mm in 0.5-mm gaps into the left striatum. After the operation, the rats were allowed to recover from the anesthesia on the heating pad. Initial inflammation due to BCG injection was mild and cleared in a short period of time [22]. Sham-operated rats (*n* = 4) were injected with 2 μL of phosphate-buffered saline (PBS).

Four weeks after the intra-cerebral injection, the *f*DTH-EAE lesion was activated with a subcutaneous (s.c.) injection of 100 μL complete Freund’s adjuvant (CFA)/saline (Sigma-Aldrich, St. Louis, MO, USA) emulsion supplemented with 1.5 mg *Mycobacterium tuberculosis* (TB) (heat-killed Mycobacterium tuberculosis-H37Ra, InvivoGen, San Diego, CA, USA) to the hind limbs of the rat. The emulsion induces the development of a focal DTH-like lesion, subsequent to BBB disruption and peripheral lymphocyte recruitment [22]. The sham-operated rats were injected s.c. with saline.

### *f*MOG-EAE, type II animal model of MS

For the *f*MOG-EAE model, anesthetized rats (*n* = 16) were injected s.c. at the base of the tail with 100 μL of rat MOG (35–55) peptide (Sigma Aldrich, St. Louis, MO, USA) emulsified in incomplete Freund’s adjuvant (IFA) (Sigma-Aldrich, St. Louis, MO, USA). Sham-operated rats (*n* = 4) were injected with IFA/saline emulsion. Three weeks later, MOG-immunized rats were operated by a stereotactic injection of 2-μL cytokine mixture in a glass micro capillary needle containing 1.45 μg of recombinant rat tumor necrosis factor-α (TNF-α, Sigma-Aldrich, St. Louis, MO, USA) and 1 μg recombinant rat interferon-γ (IFN-γ, Sigma-Aldrich, St. Louis, MO, USA) into the corpus callosum to form a focal EAE lesion. (Stereotactic experiments were conducted as described above.) The sham-operated rats were injected with 2 μL of PBS.

### MRI

After inducing *f*DTH-EAE and *f*MOG-EAE, the rats were MR imaged on day 2 post-induction (*n* = 4 and *n* = 8, respectively). Separate groups of rats were MR imaged on day 13 post-induction (*n* = 4 and *n* = 8, respectively). MRI was performed using clinical Philips Achieva 3T device (Philips Medical Systems, Koninklijke, The Netherlands). Anesthesia was initiated with a mixture of 4–5% isoflurane and air (500-700 mL/min) while the rats were on the heating pad. A cannula was set into the tail vein for the administration of 100 μL of the Gd-contrast agent (DOTAREM 279.3 mg/mL, Guerbet, Roissy, France) 10 min prior to the post T1-weighted imaging to assess BBB permeability. For MRI, the rats were placed in a rat-dedicated brain coil (Rat Brain Array 4, RAPID Biomedical GmbH, Rimpar, Germany) and anesthesia was maintained with 2–2.5% isoflurane (400–500 mL/min). Body temperature was kept at + 37 °C by an external heating system (RAPID Air Heating Control, RAPID Biomedical GmbH, Rimpar, Germany).

First, scout images were obtained to define the brain area to be scanned. Pre and post T1-weighted spin-echo sequences were acquired using the repetition time (TR) of 600 ms and echo time (TE) of 14 ms. The T2-weighted images were obtained using the turbo spin echo (TSE) sequence with TR 4000 ms, TE 75 ms and TSE factor 10. The field of view (FOV) was 50 mm × 50 mm × 17.6 mm for the T1-weighted images and 45 mm × 45 mm × 21.6 mm for the T2-weighted images. The final voxel size was 0.15 mm × 0.15 mm × 0.8 mm for the T1-weighted images and 0.14 mm × 0.14 mm × 1.2 mm for the T2-weighted images. MRI data were analysed using Inveon Research Workplace v4.1 software (Siemens Medical Solutions, Malvern, PA, USA). Post T1-weighted and T2-weighted images were first thresholded according to the signal intensity in non-injected brain hemispheres, as described previously [18]. Post T1-weighted MR images were analysed by defining regions of interest (ROIs) on the area of Gd-enhancement and ROIs of the same size on the contralateral intact brain hemisphere. Areas of increased hyper-intensity from T2-weighted images were analysed using the same protocol. The results were expressed as lesion to contralateral intact brain hemisphere ratios.

### Ex vivo autoradiography with VAP-1 targeted radiotracer

A cyclic peptide with disulfide-bridged cysteines, CARLSLSWRGLTLCPSK, consisting of residues 283−297 from the Siglec-9 and having 8-amino-3,6-dioxaoctanoyl linker (polyethylene glycol derivative) between DOTA and peptide (Peptide Speciality Laboratories, Heidelberg, Germany) was labelled with ^68^Ga as previously described [23], with the exception that 3 nmol of DOTA-Siglec-9 was used for each labelling batch. This Siglec-9-derived cyclic peptide is known to bind to VAP-1 [24]. The radiochemical purity of ^68^Ga-DOTA-Siglec-9 was determined by radiodetector-coupled high-performance liquid chromatography (radio-HPLC) (Jupiter C18 column, 4.6 × 150 mm, 300 Å, 5 μm; Phenomenex, Torrance, CA, USA). Specific radioactivity at the end of synthesis was 180 ± 140 MBq/nmol and radiochemical purity was > 95% throughout the study.

The *f*DTH-EAE, *f*MOG-EAE, and sham-operated rats were intravenously (i.v.) injected with ^68^Ga-DOTA-Siglec-9 (34 ± 5.1 MBq) on day 3 (*n* = 4, *n* = 7 and *n* = 4, respectively) and on day 14 (*n* = 4, *n* = 8 and *n* = 4, respectively) after the disease activation, and sacrificed 45 min after the tracer injection for digital ex vivo autoradiography analysis. Anti-VAP-1 polyclonal antibody was injected i.v. 10 min prior to sacrifice in order to allow visualization of luminal VAP-1 by immunofluorescence. Under deep anesthesia (isoflurane 4%, 700 mL/min), blood was collected via cardiac puncture from the left ventricle. After sacrifice, the brains were collected, frozen and sectioned into 20- and 10-μm slices with a cryomicrotome (Leica CM 3050 S cryostat, Leica Biosystems, Nussloch, Germany) for the purposes of hematoxylin-eosin (H&E) staining, immunohistochemistry and immunofluorescence. The entire lesion in the brain was cut on positively charged slides (Superfrost Ultra Plus, Thermo Fisher, Pittsburgh, PA, USA), briefly air-dried and exposed to imaging plates (BAS-TR2025, Fuji Photo Film Co, Ltd., Tokyo, Japan) for approximately 140 min (i.e. two physical half-lives of ^68^Ga) in order to collect data for autoradiography. The plates were scanned with a phosphor imaging scanner (BAS-5000, Fuji, Tokyo, Japan; 25 μm internal resolution).

The digital autoradiography data were analysed with AIDA Image analyzer v4.55 software (Raytest Isotopenmessgeräte GmbH, Straubenhardt, Germany) by defining the ROIs in the lesion hemisphere and the contralateral intact hemisphere for the determination of count densities of ^68^Ga-DOTA-Siglec-9 binding as photostimulated luminescence per square millimeter (PSL/mm^2^). ROIs were also defined on the cortical areas to determine the extent of the inflammation surrounding the lesion. Results were expressed as bound-to-free ratio, which was calculated according to the following equation:

$$ \left({\left(\mathrm{PSL}/{\mathrm{mm}}^2\right)}_{\mathrm{Lesion}}\hbox{--} {\left(\mathrm{PSL}/{\mathrm{mm}}^2\right)}_{\mathrm{Contralateral}}\right)/{\left(\mathrm{PSL}/{\mathrm{mm}}^2\right)}_{\mathrm{Contralateral}} $$

The bound-to-free ratio was individually calculated from each brain slice and averaged across all individual bound-to-free ratios.

In addition, the uptake of the radiotracer in brain was calculated as a percentage of injected radioactivity dose (%ID) according to the following equation:

$$ \left(\mathrm{Radioactivity}\ \mathrm{of}\ \mathrm{the}\ \mathrm{brain}\ \left(\mathrm{MBq}\right)/\mathrm{Injected}\ \mathrm{radioactivity}\ \mathrm{dose}\ \left(\mathrm{MBq}\right)\right)\ast 100 $$

### Histology, immunofluorescence and immunohistochemistry

In addition to the ^68^Ga-DOTA-Siglec-9 ex vivo autoradiography, sequential brain cryosections were cut for histology, immunofluorescence and immunohistochemistry. Acetone-fixed or formalin-fixed sections were stained with H&E or Luxol fast blue (LFB) with cresyl violet counterstain, respectively, using the standard procedures. For the purpose of visualizing the activated microglia with OX-42 staining, the sections were post-fixed with periodate-lysine-paraformaldehyde (PLP) for 20 min [25] and washed with PBS. PLP fixation was followed by a quenching step, in which the endogenous peroxidase was blocked by incubation of 1% H_2_O_2_ in methanol for 20 min. Normal 10% horse serum diluted with PBS was used to block unspecific binding of the primary antibody in the tissue (Vector Laboratories Inc., Burlingame, Colorado, CO, USA). The primary antibody (anti-OX-42, Abcam, Cambridge, UK) was added for overnight incubation (16 h). Incubation was followed by the addition of a secondary antibody (anti-mouse Ig). An ABC-kit was used to form avidin-biotin complexes attached to the secondary antibodies (Vectastain, Vector Laboratories). 3,3-diaminobenzidine (DAB) was used as a chromogen. The sections were counterstained with hematoxylin and mounted with ProLong Gold antifade reagent (Life Technologies P36930).

For IgG and neurofilament protein (NFP) immunohistochemical staining, the cryosections were fixed with 4% paraformaldehyde and washed with PBS supplemented with 0.05% Tween-20. Quenching step and blocking of unspecific binding were performed in the same way as in OX-42 staining described above. Then, the sections were incubated overnight with anti-NFP antibody (M0762, clone 2F11, Dako, Santa Clara, CA, USA). Incubation was followed by the addition of a secondary anti-rat Ig antibody (Dako, K4001). For assessment of IgG leakage, the sections were incubated with only anti-rat Ig (Dako, K4003). After DAB reaction, the sections were counterstained with hematoxylin and mounted with ProLong Gold antifade reagent (Life Technologies P36930).

The stained sections were evaluated under a light microscope. H&E, LFB, IgG, NFP and OX-42 stainings were scanned using Pannoramic 250 F scanner (3D Histech, Budapest, Hungary) and analysed with Pannoramic viewer (3D Histech, Budapest, Hungary). The area of activated microglia was determined by defining ROIs on the estimated area of increased microglial activation. The lymphocyte infiltration was quantified by counting lymphocytes from four non-overlapping areas of approximately 0.6 mm^2^ within the lesion as observed in H&E stained sections. The areas were chosen according to the highest density of recruited lymphocytes within the parenchyma, and the results were expressed as a lymphocyte count per mm^2^. The amount of demyelination was scored from 0 to 3 based on LFB staining. The percent area of altered NFP expression or IgG leakage were determined by defining ROIs on four sections with positive immunostained areas and averaged for each rat. This area was then divided by the averaged total brain area per section for each group.

For VAP-1 immunofluorescence staining, the sections were incubated with fluorescein isothiocyanate (FITC) conjugated (Sigma F1262; 1:40 + 5% normal rat serum) goat anti-rabbit IgG secondary antibody (Life Technologies A11034). The signal was amplified with anti-Fluorescein-488 (Invitrogen A11096, 10 μg/mL) and then mounted with ProLong Gold antifade reagent (Life Technologies P36930). Alternatively, polyclonal anti-VAP-1 antibody (produced in rabbits against recombinant human VAP-1 but recognizing also rat VAP-1) was applied for 30 min to detect intracellular and surface-bound VAP-1 [26].

For ICAM-1 and P-selectin immunofluorescence staining, the sections were first fixed with ice-cold acetone for 3 min and washed with PBS. Then, the samples were incubated either with primary anti-ICAM-1 antibody (Santa Cruz sc-7891; 1:20 dilution) or with anti-P-selectin antibody (Santa Cruz sc-6943; 1:100 dilution) for 30 min in room temperature. Thereafter, the sections were incubated with FITC-conjugated goat anti-rabbit secondary antibody (Sigma F1262-2ML; 1:100 dilution). The signal was amplified with anti-Fluorescein-488 (Invitrogen A11096, 5 μg/mL), and the sections were mounted as described above.

Immunofluorescence stainings were evaluated with Olympus BX 60 microscope (Olympus Corporation, Hamburg, Germany) and captured with Pannoramic MIDI scanner (3D Histech, Budapest, Hungary) or with a Zeiss LSM780 confocal microscope (Carl Zeiss MicroImaging GmbH, Jena, Germany). Tissue auto-fluorescence was distinguished from specific VAP-1 staining using the spectral scan mode and subsequent linear un-mixing based on reference spectra. The degree of VAP-1, ICAM-1 and P-selectin positivity were graded from − (score 0), no positivity; + (score 1), faint positivity with a few positive vessels detected – less than five vessels/× 40 field; +++ (score 3), strong positivity of many vessels (11–20 vessels/× 40 field). The rating of ++ (score 2), 6–10 vessels/× 40 field, was given to samples falling between categories + and +++ [7].

### Statistical analyses

All results are presented as mean ± standard deviation with two significant figures. Statistical analyses were conducted using Graph Pad Prism v5.01 software (Graph Pad Software Inc., La Jolla, CA, USA). For comparisons between the groups, Student’s *t* test for paired data was used. *P* values less than 0.05 were considered statistically significant.

## Results

### Rat EAE lesions can be assessed using 3-T MRI

**Fig. 2:**
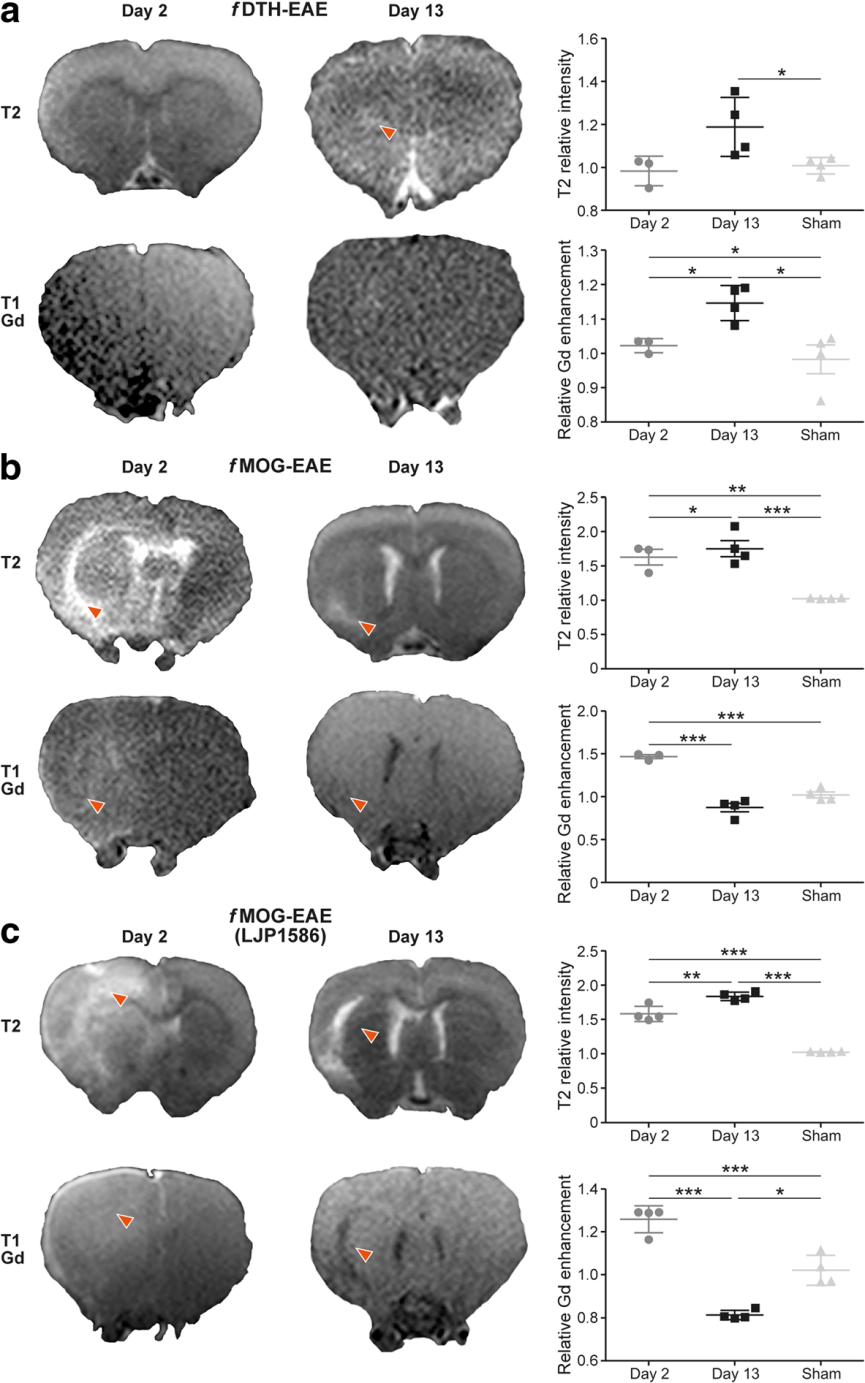
Traditional and LJP1586-treated EAE lesions can be observed with clinical 3-T MRI using a rat-dedicated brain coil. (**a**) Representative T2-weighted and Gd-enhanced T1-weighted coronal in vivo MR images and corresponding quantitative data of *f*DTH-EAE (*n* = 4) (**b**) Representative T2-weighted and Gd-enhanced T1-weighted coronal in vivo MR images and corresponding quantitative data of *f*MOG-EAE (*n* = 4). (**c**) Representative T2-weighted and Gd-enhanced T1-weighted coronal in vivo MR images and corresponding quantitative data of LJP1586-treated *f*MOG-EAE (*n* = 4). Red arrows indicate lesion sites. T2-weighted MR images display inflammation in hyper-intense areas and Gd-enhanced T1-weighted images reveal BBB status during the course of the disease. Sham-operated rats (*n* = 4) were used for comparison. *P < 0.05, **P < 0.01, ***P < 0.001

**Fig. 3:**
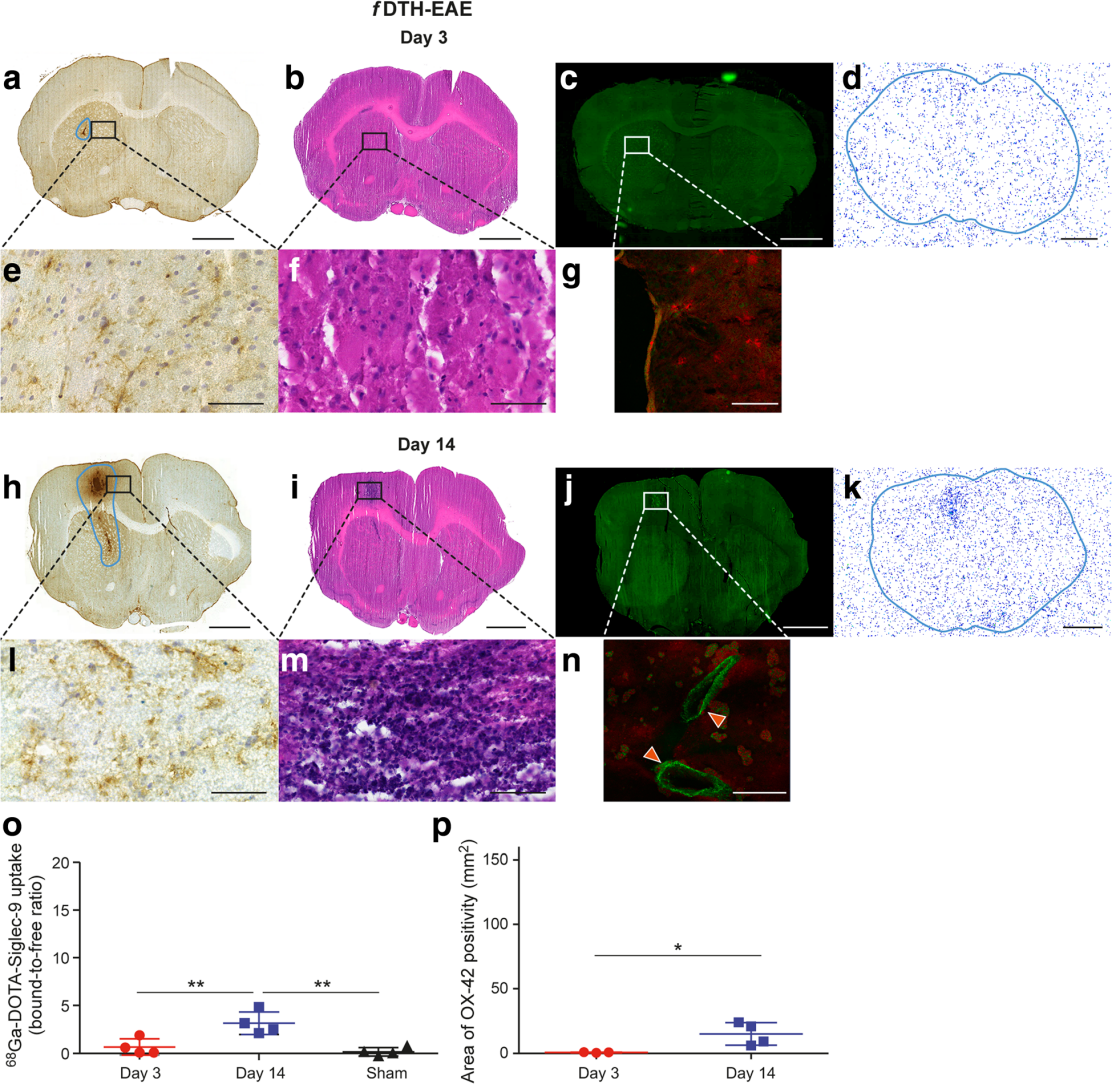
Immunohistochemistry, histology, immunofluorescence and autoradiography of *f*DTH-EAE rat brain sections with supporting quantitative data. (**a**, **h**) OX-42 immunohistochemical staining of activated microglia (the contours define the drawn ROIs used for quantification). (**b**, **i**) H&E staining. (**c**, **j**) Anti-VAP-1 immunofluorescence staining with secondary antibody captured with Pannoramic MIDI scanner. (**d**, **k**) VAP-1 targeted ^68^Ga-DOTA-Siglec-9 ex vivo autoradiography. Autoradiography brain sections are outlined according to the corresponding sections in immunohistochemistry. Low power scale bars = 2 mm. High power (**e**, **l**) OX-42, (**f**, **m**) H&E, (**g**, **n**) anti-VAP-1 immunofluorescence staining with secondary antibody captured with confocal microscope. Tissue auto-fluorescence signal (red colour) is separated from specific VAP-1 staining (green) using the spectral imaging followed by linear un-mixing based on the reference spectra. High power scale bars = 50 μm. Red arrows point to VAP-1 positive vessels in brain vasculature. Quantification of autoradiography data (**o**) and area of activated microglia (**p**) for *f*DTH-EAE. *P < 0.05, **P < 0.01, ***P < 0.001

Intra-striatally injected BCG suspension and subsequent peripheral activation of *f*DTH-EAE lesions resulted in the following characteristics of a typical active MS-like lesion at days 13−14 after disease activation: (1) BBB breakdown visualized with Gd-enhanced T1-weighted MRI, (2) hyper-intense areas in T2-weighted MR images, (3) well-defined OX-42 positivity and (4) IgG leakage as detected by immunohistochemistry. In contrast, for the pre-active lesions at day 2−3, the MRI showed intact BBB with no visible lesion, whereas OX-42 positivity was present in low amounts (Figs. 2a and 3). Minimal anti-Ig positive staining supported the MRI findings (Fig. 6a, d).

**Fig. 4:**
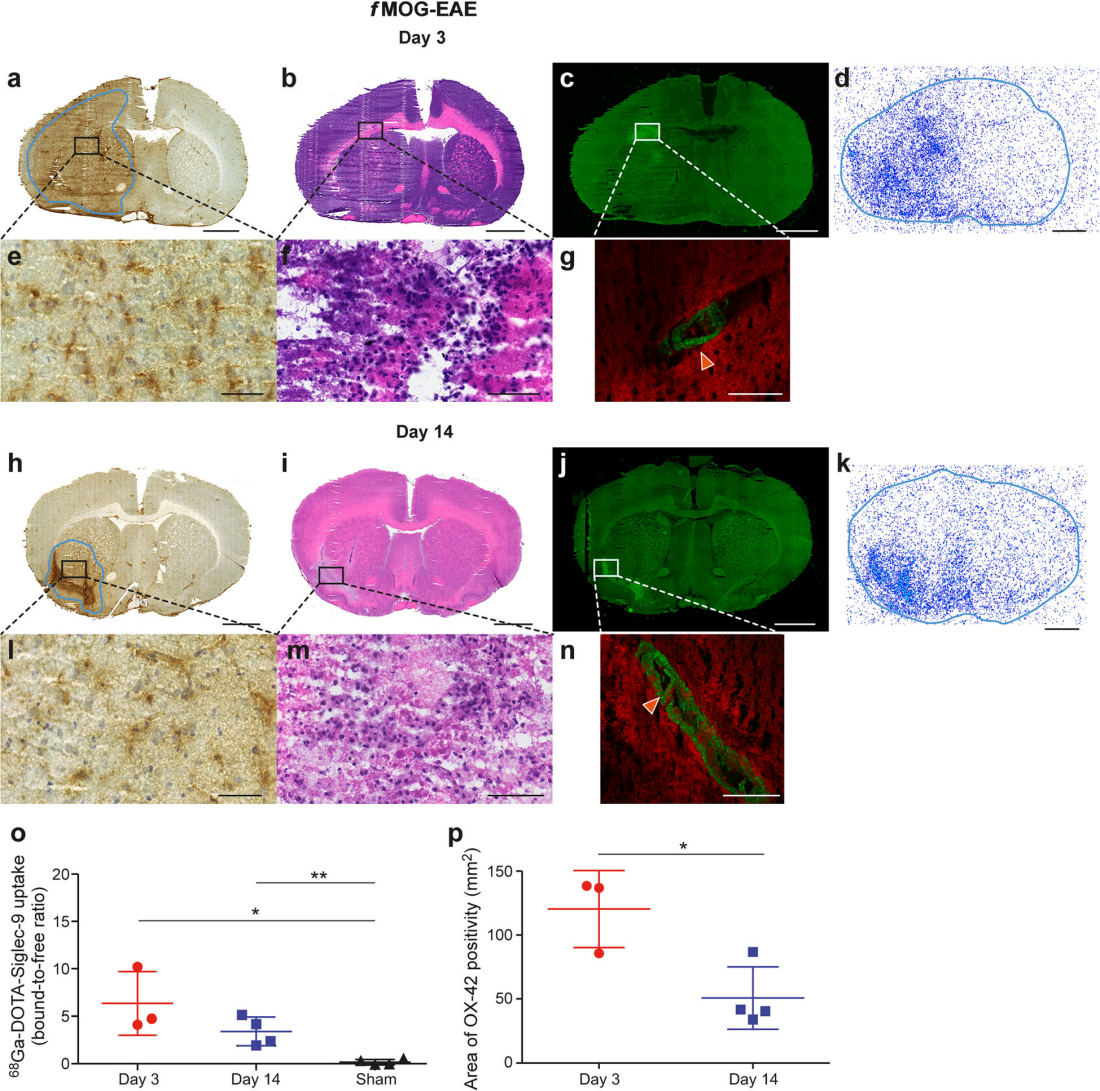
Immunohistochemistry, histology, immunofluorescence and autoradiography of *f*MOG-EAE rat brain sections with supporting quantitative data. (**a**, **h**) OX-42 immunohistochemical staining of activated microglia (the contours define the drawn ROIs used for quantification). (**b**, **i**) H&E staining. (**c**, **j**) Anti-VAP-1 immunofluorescence staining with secondary antibody captured with Pannoramic MIDI scanner. (**d**, **k**) VAP-1 targeted ^68^Ga-DOTA-Siglec-9 ex vivo autoradiography. Autoradiography brain sections are outlined according to the corresponding sections in immunohistochemistry. Low power scale bars = 2 mm. High power (**e**, **l**) OX-42, (**f**, **m**) H&E, (**g**, **n**) anti-VAP-1 immunofluorescence staining with secondary antibody captured with confocal microscope. Tissue auto-fluorescence signal (red colour) is separated from specific VAP-1 staining (green) using the spectral imaging followed by linear un-mixing based on the reference spectra. High power scale bars = 50 μm. Red arrows point to VAP-1 positive vessels in brain vasculature. Quantification of autoradiography data (**o**) and area of activated microglia (**p**) for *f*MOG-EAE. *P < 0.05, **P < 0.01, ***P < 0.001

For *f*MOG-EAE lesions, MOG-immunization followed by an injection of cytokine mixture into corpus callosum resulted in an acute, widespread and diffuse inflammation that was visible in T2-weighted MR images and accompanied by BBB breakdown revealed by Gd-enhanced MRI and anti-IgG staining already 2 days after the activation (Figs. 2b and 6b, d). OX-42 staining revealed large areas of activated microglia during the acute phase (day 3) of the *f*MOG-EAE lesion development (Fig. 4). As expected, a reduction in the T2 hyper-intense and Gd-enhancement area was observed at 13 days after the disease activation in T2-weighted and T1-weighted Gd-enhanced images as well as decrease in anti-IgG staining positivity, thus indicating improvement in BBB status after the initial damage (Figs. 2b and 6b, d). The area of activated microglia was significantly reduced at day 14 as compared to day 3 (*P* = 0.0024, Fig. 4p).

In general, the areas of Gd-enhancement in both *f*DTH-EAE and *f*MOG-EAE correlated well with the areas of T2-weighted hyper-intensity. Also, the size and location of EAE lesions in MR images correlated well with ^68^Ga-DOTA-Siglec-9 binding.

### VAP-1 expression is associated with the development of inflammatory brain lesions in EAE rat models

In the case of *f*DTH-EAE, VAP-1 expression emerged after the disease development from the pre-active phase to the active inflammatory phase. In early pre-active lesions, only a very weak ex vivo autoradiography signal of ^68^Ga-DOTA-Siglec-9 was detected, although few OX-42 positive cells were already present (Fig. 3a, d). Immunofluorescence staining at day 3 revealed occasional VAP-1 positivity in brain parenchyma, although the translocation of VAP-1 to endothelial cell surface was absent. The pre-active lesions did not show evidence of demyelination, although the NFP staining pattern was slightly altered (Fig. 6a, f). During the acute inflammatory phase (at day 14), luminal VAP-1 and OX-42 positivity were detected by immunofluorescence and immunohistochemistry stainings (Fig. 3). Immunofluorescence staining indicated a visually higher signal for VAP-1 and translocated VAP-1 at day 14 compared to day 3 (Fig. 3 and Table 1). The immunofluorescence signal of most endothelial cells in *f*DTH-EAE resembled the staining pattern of regular thinner flat-walled morphology, which is typically found in small venules although some vessels showed signs of transformation to the plump morphology, typical for HEV-like venules at sites of inflammation [6, 7]. OX-42 immunohistochemistry for *f*DTH-EAE rats showed a significant increase (*P* = 0.039) in OX-42 positivity at day 14 as compared to the earlier phase at day 3 (Fig. 3p). During active inflammatory phase, the NFP expression pattern was similar as seen in pre-active lesions, but the LFB staining now revealed mild demyelination around the lesions (Fig. 6a, e, f) though not significantly different from day 3 (*P* = 0.13). Binding of ^68^Ga-DOTA-Siglec-9 at the site of *f*DTH-EAE lesion was significantly higher 14 days after disease activation as compared to the sham-operated rats (Fig. 3o; 3.2 ± 0.59 vs. 0.16 ± 0.22 PSL/mm^2^, *P* = 0.0031) and the *f*DTH-EAE rats sacrificed at day 3 (Fig. 3o; *P* = 0.014). ^68^Ga-DOTA-Siglec-9 binding depicted no significant difference between *f*DTH-EAE rats (day 3) and sham-operated rats (*P* = 0.32).

**Table 1:** Semiquantitative immunofluorescence findings of rat brains

Experimental group	ICAM-1	P-selectin	VAP-1
*f*DTH-EAE, Day 3	+	−	−
*f*DTH-EAE, Day 14	+	−	+
*f*MOG-EAE, Day 3	++	−	++
*f*MOG-EAE, Day 14	++	−	++
*f*MOG-EAE (LJP1586), Day 3	−	−	−
*f*MOG-EAE (LJP1586), Day 14	+++	−	−

−, no positivity; +, faint positivity with a few positive cells detected; ++, moderate positivity; +++, strong positivity of many cells

**Fig. 5:**
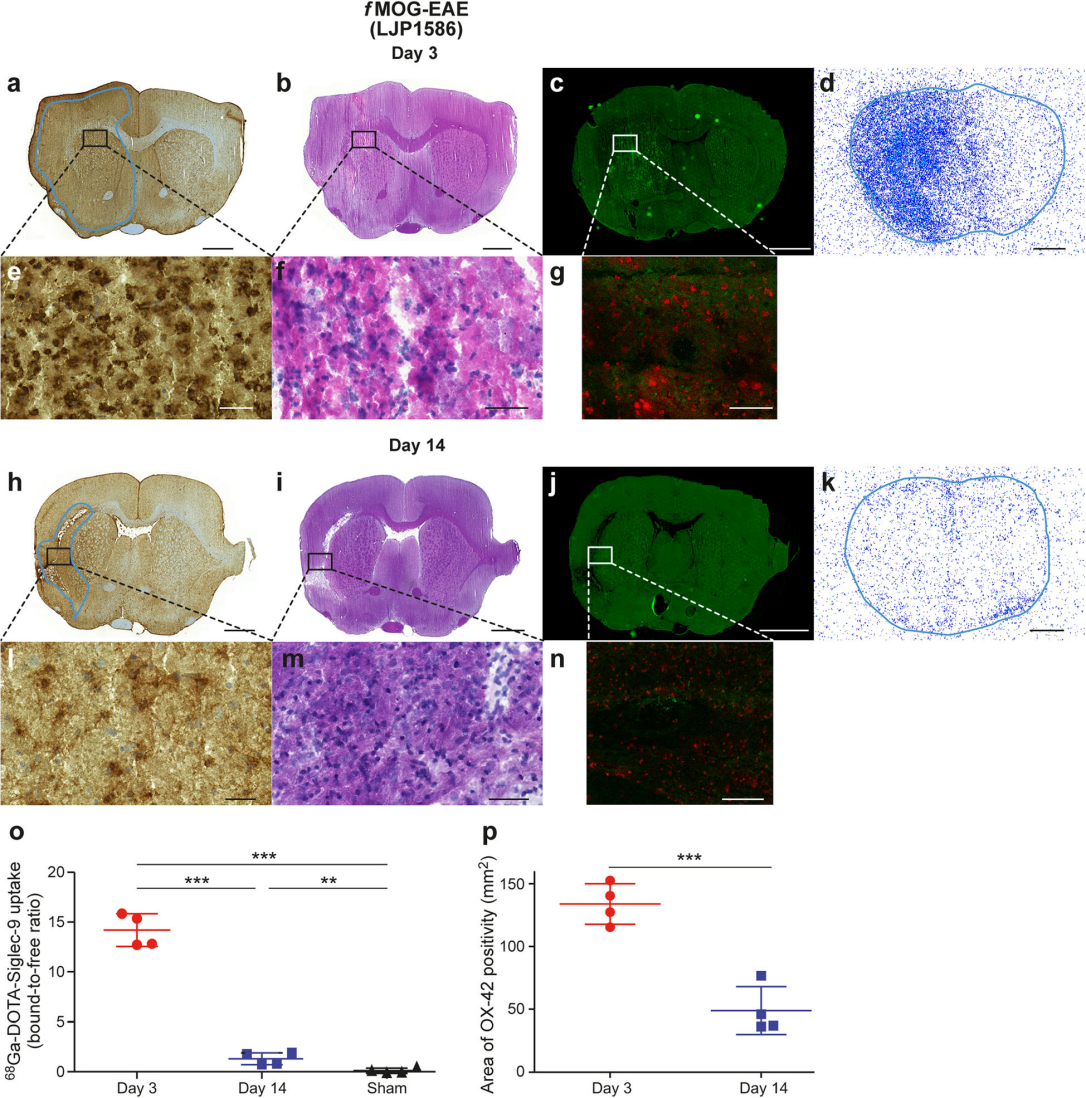
Immunohistochemistry, histology, immunofluorescence and autoradiography of LJP1586-treated *f*MOG-EAE rat brain sections with supporting quantitative data. (**a**, **h**) OX-42 immunohistochemical staining of activated microglia (the contours define the drawn ROIs used for quantification). (**b**, **i**) H&E staining. (**c**, **j**) Anti-VAP-1 immunofluorescence staining with secondary antibody captured with Panoramic MIDI scanner. (**d**, **k**) VAP-1 targeted ^68^Ga-DOTA-Siglec-9 ex vivo autoradiography. Autoradiography brain sections are outlined according to the corresponding sections in immunohistochemistry. Low power scale bars = 2 mm. High power (**e**, **l**) OX-42, (**f**, **m**) H&E, (**g**, **n**) anti-VAP-1 immunofluorescence staining with secondary antibody captured with confocal microscope. Tissue auto-fluorescence signal (red colour) is separated from specific VAP-1 staining (green) using the spectral imaging followed by linear un-mixing based on the reference spectra.. High power scale bars = 50 μm. Red arrows point to VAP-1 positive vessels in brain vasculature. Quantification of autoradiography data (**o**) and area of activated microglia (**p**) for LJP1586 treated *f*MOG-EAE. *P < 0.05, **P < 0.01, ***P < 0.001

**Fig. 6:**
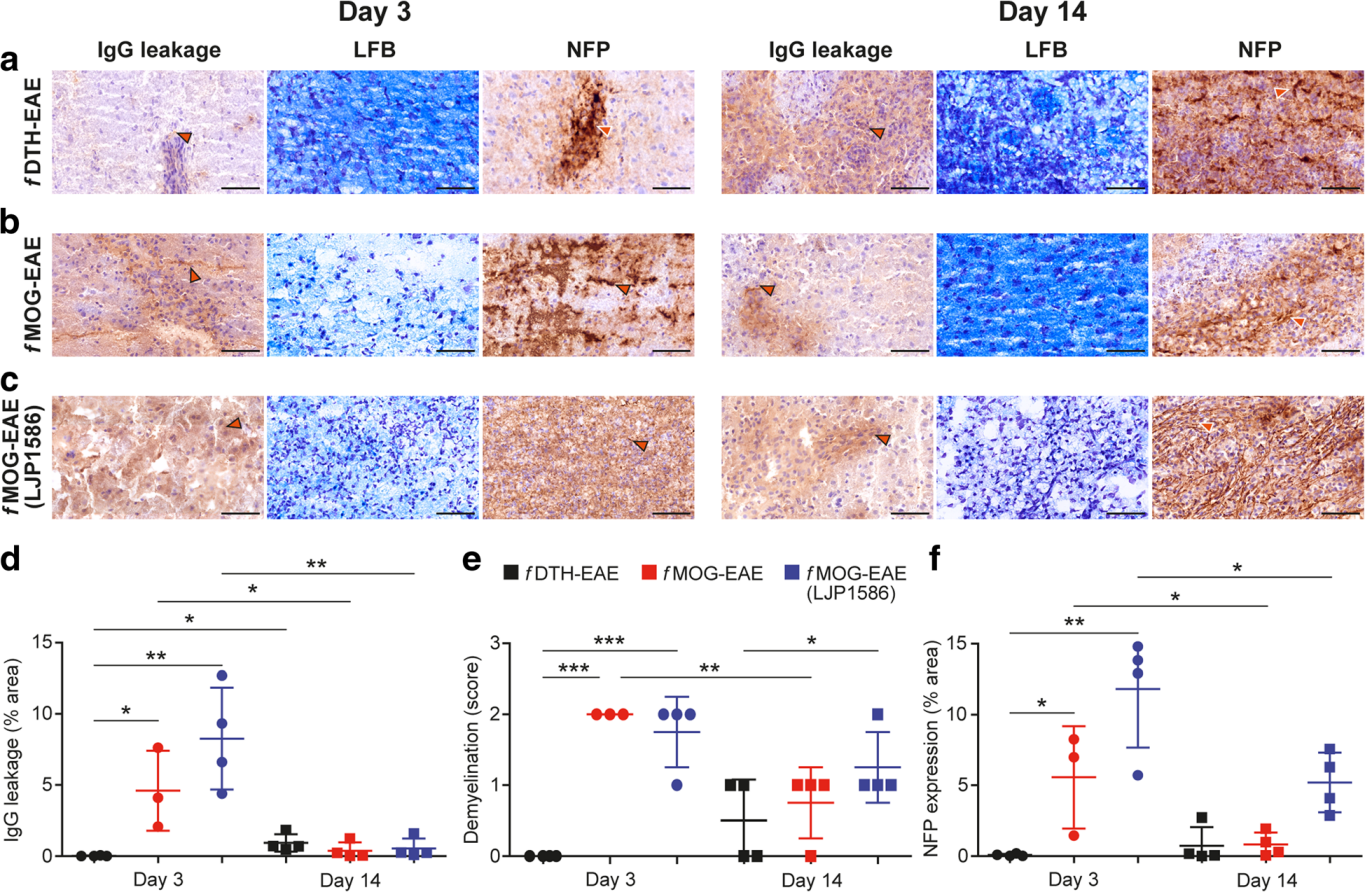
Histopathological comparison of the studied EAE rat models. Immunohistochemical stainings of (**a**) *f*DTH-EAE, (**b**) *f*MOG-EAE and (**c**) LJP1586 treated *f*MOG-EAE rat brains. Quantification of (**d**) IgG leakage, (**e**) demyelination and (**f**) altered NFP expression. Red arrows point to staining positivity. High power scale bars = 50 μm. *P < 0.05, **P < 0.01, ***P < 0.001

In the case of *f*MOG-EAE, expression patterns of VAP-1 were different during the various stages of lesion activity, as compared to *f*DTH-EAE (Fig. 4). In *f*MOG-EAE lesions, ^68^Ga-DOTA-Siglec-9 binding was significantly higher in the ipsilateral hemisphere, as compared to the sham-operated rats, both at day 3 (*P =* 0.017) and day 14 (*P* = 0.0055) after the disease induction (Fig. 4d, k, o). At day 14, the ^68^Ga-DOTA-Siglec-9 binding in the ipsilateral hemisphere was at the same level as at day 3 (*P* = 0.17). Immunofluorescence staining showed high amount of luminal VAP-1 in the lesion area during the acute phase of the disease (day 3; Fig. 4c, g and Table 1) and during the remission phase of the disease (day 14; Fig. 4j, n and Table 1). The endothelial cells in the lesion area in *f*MOG-EAE brains displayed characteristics of endothelial cells typically observed in HEV-like vessels [6]. In contrast to ^68^Ga-DOTA-Siglec-9 binding, OX-42 staining showed a significant difference between day 3 and day 14 (*P* = 0.019) in *f*MOG-EAE rats, with more prominent OX-42 positivity on day 3 (Figs. 4 and 5). LFB staining indicated moderate loss of myelin at day 3 and no difference in demyelination score at day 14 compared to day 3 (Fig. 6b, e; *P* = 0.72). However, there was a significant decrease in area of NFP expression at remission phase compared to day 3 (Fig. 6b, f; *P* = 0.048). In conclusion, despite of restauration of the BBB at day 14 after activation, luminal VAP-1 expression remained in the *f*MOG-EAE lesion vasculature as detected by VAP-1 targeted ^68^Ga-DOTA-Siglec-9 ex vivo autoradiography and immunofluorescence detection of i.v. injected anti-VAP-1 antibody.

### Expression of endothelial homing determinants within the EAE lesions post VAP-1 inhibitor treatment

**Fig. 7:**
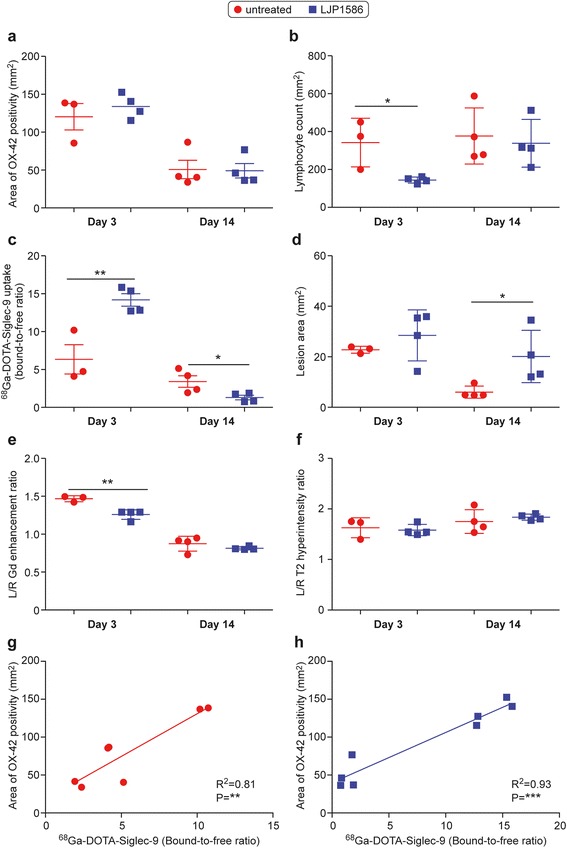
Comparison of immunohistochemistry, histology and autoradiography data between *f*MOG-EAE and LJP1586 treated *f*MOG-EAE. (**a**) Area of microglial activation, (**b**) lymphocyte count densities, (**c**) ^68^Ga-DOTA-Siglec-9 ex vivo autoradiography, (**d**) lesion area, (**e**) signal changes in T1 and T2 weighted MR images and correlation between OX-42 positivity and ^68^Ga-DOTA-Siglec-9 ex vivo autoradiography in (**f**) LJP1586 treated and (**g, h**) untreated *f*MOG-EAE. *P < 0.05, **P < 0.01, ***P < 0.001

Treatment with small molecular VAP-1 inhibitor LJP1586 reduced the amount of lymphocytes in acute inflammatory phase of *f*MOG-EAE lesions by 58% when compared with untreated rats (day 3; inhibitor, 144 ± 16 counts/mm^2^ vs. untreated, 342 ± 128 counts/mm^2^; *P* = 0.026; Fig. 7b), but the lymphocyte infiltration was restored in the remission phase (day 14; inhibitor, 338 ± 126 counts/mm^2^ vs. untreated, 377 ± 148 counts/mm^2^; *P* = 0.70; Fig. 7b). In contrast, despite the LJP1586 treatment, the amount of OX-42-positive cells in CNS lesions remained unchanged (Fig. 7a). LFB immunohistochemistry showed moderate demyelination in LJP1586 treated *f*MOG-EAE rats at both day 3 and day 14, but no difference between untreated and treated *f*MOG-EAE rats at either disease phase (Fig. 6c, e; *P* = 0.72 at day 3, *P* = 0.54 at day 14). Similarly to untreated *f*MOG-EAE, NFP staining showed significant decrease in area of altered NFP expression in LJP1586 treated rats at day 14 compared to day 3 (*P* = 0.030), but no difference between untreated and treated rats (Fig. 6c, f; *P* = 0.093 at day 3, *P* = 0.086 at day 14).

**Fig. 8:**
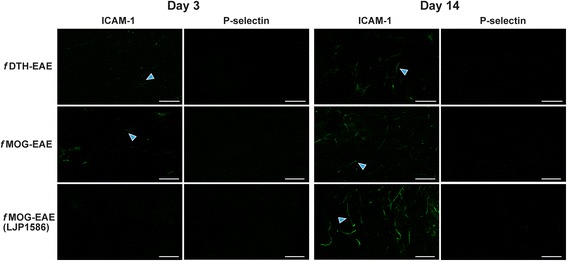
Immunofluorescence showing ICAM-1 and P-selectin expression patterns in brain vasculature during the time course of disease in *f*DTH-EAE, *f*MOG-EAE and LJP1586-treated *f*MOG-EAE. The LJP1586 treatment reduced the expression of ICAM-1 only in the acute phase of the disease. Untreated *f*DTH-EAE rat brains showed weak ICAM-1-positivity in vessels of early pre-active lesions and acute inflammatory lesions. P-selectin-detecting stainings remained negative throughout the studies. Rat brains that had EAE lesions at day 3 or day 14 were stained with ICAM-1 and P-selectin using immunofluorescence. Blue arrows point to ICAM-1 positivity in brain vessels. High power scale bars = 50 μm

The semiquantitative results from VAP-1, ICAM-1 and P-selectin stainings are presented in Table 1. In *f*MOG-EAE, the LJP1586 treatment decreased the expression of ICAM-1 in the acute phase, but it was restored during the restauration of BBB integrity to the levels of untreated *f*MOG-EAE as observed by semi-quantitative analysis (Fig. 8 and Table 1). Untreated *f*DTH-EAE rat brains showed weak ICAM-1-positivity in vessels of early pre-active lesions and acute inflammatory lesions. P-selectin-detecting staining remained negative throughout the studies (Fig. 8 and Table 1).

The expression of VAP-1 in the lesion vasculature tended to decrease during both the acute (day 3; Fig. 5c, g, Table 1) and the remission phases of the disease (day 14; Fig. 5 j, n, Table 1) subsequent to LJP1586 treatment. Notably, when the anti-VAP-1 antibody is i.v. injected, the secondary antibody applied on tissue sections detects endothelial cell surface VAP-1. In the same way, i.v. administered radioligand ^68^Ga-DOTA-Siglec-9 also detects only luminal VAP-1. In the acute phase, the uptake of luminal VAP-1 targeted radiotracer, ^68^Ga-DOTA-Siglec-9, was significantly higher in LJP1586-treated *f*MOG-EAE CNS lesion hemisphere compared with the untreated rats (day 3; inhibitor, 14 ± 1.6 bound-to-free ratio vs. untreated, 6.3 ± 3.3 bound-to-free ratio; *P* = 0.0088; Fig. 7c). However, in the remission phase, the binding of ^68^Ga-DOTA-Siglec-9 was lower compared to untreated (day 14; inhibitor, 1.3 ± 0.59 bound-to-free ratio vs. untreated, 3.4 ± 1.5 bound-to-free ratio; *P* = 0.041; Fig. 7c). Interestingly, the CNS lesion was significantly larger at day 14 in the LJP1586-treated rats compared to untreated (inhibitor, 20.1 ± 5.2 mm^2^ vs. untreated, 6.0 ± 1.2 mm^2^, *P* = 0.039, Fig. 7d). Ex vivo gamma counting indicated significantly increased uptake of ^68^Ga-DOTA-Siglec-9 in both the untreated (0.026 ± 0.0031%ID) and LJP1586-treated *f*MOG-EAE rats brain (0.026 ± 0.012%/ID) at day 3 post-activation as compared to the sham-operated controls (0.011 ± 0.0020 %ID, *P* = 0.00011). Both VAP-1 immunofluorescence and autoradiography showed an increased signal in densely OX-42 positive cells infiltrated areas in untreated *f*DTH-EAE and *f*MOG-EAE (Figs. 3 and 4). This OX-42 positive area correlated with VAP-1 autoradiography in LJP1586-treated (Fig. 7g; *R*^2^ = 0.93, *P* = 0.0010) and untreated *f*MOG-EAE (Fig. 7h; *R*^2^ = 0.81, *P* = 0.0061).

The LJP1586 treatment induced a significant reduction in Gd enhancement during the acute phase (day 2) of the inflammation (Figs. 2c and 7e; *P* = 0.0041), whereas hyper-intense T2-weighted MRI indicated no difference at day 2 or day 13 in *f*MOG-EAE (Fig. 7f). As opposed to previous MRI findings, LJP1586 treatment had no effect on area of IgG leakage on *f*MOG-EAE rats (Fig. 6c, d; *P* = 0.21 at day 3, *P* = 0.73 at day 14).

### Choroid plexus and meningeal vessels express occasional VAP-1 positivity in EAE rats

**Fig. 9:**
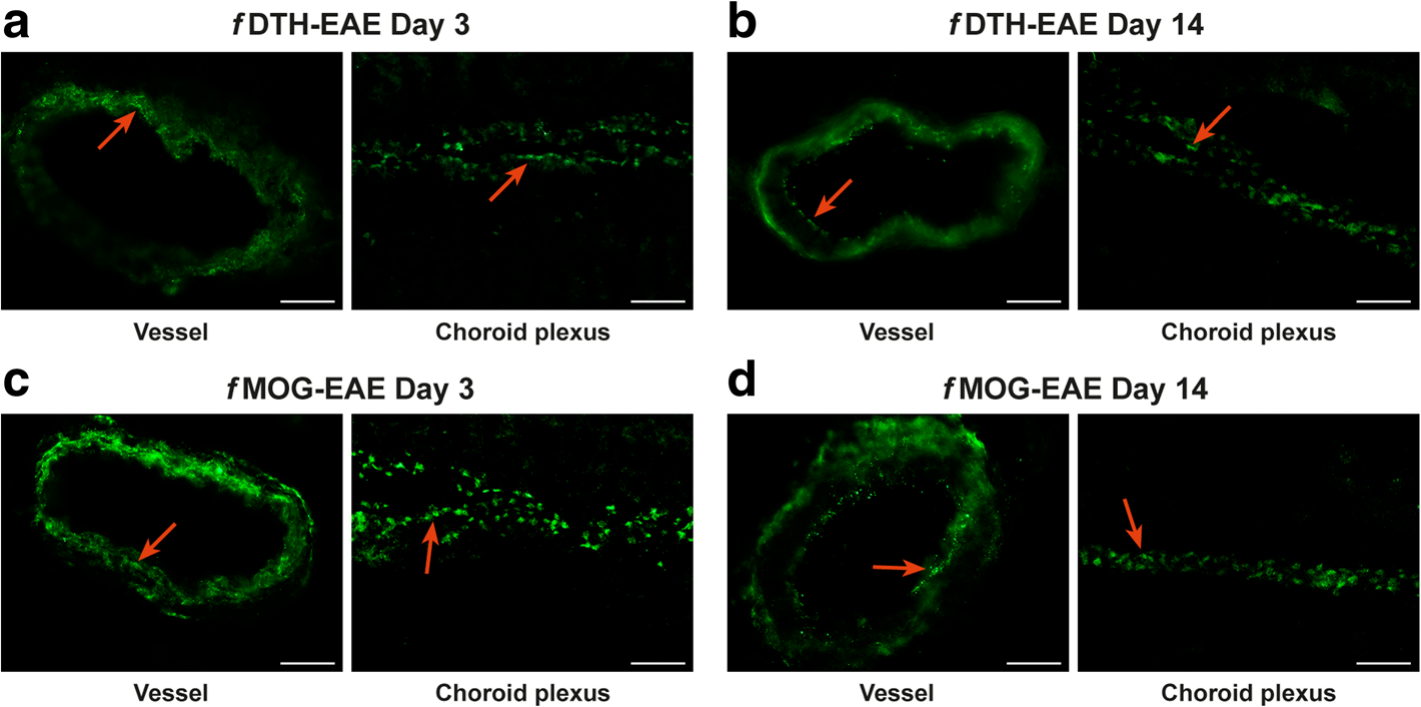
Cross-sections of (**a**, **b**) *f*DTH-EAE and (**c**, **d**) *f*MOG-EAE meningeal vessels and choroid plexus stained with polyclonal anti-VAP-1 antibody. Red arrows point to VAP-1 positivity in choroid plexus or in vessels. High power scale bar = 50 μm

To determine the VAP-1 expression outside of the lesion vasculature, a subset of EAE brain cryosections were immunohistochemically stained with a polyclonal anti-VAP-1 antibody. Figure 9 shows *f*DTH-EAE and *f*MOG-EAE meningeal vessels and choroid plexus. Positive staining of VAP-1 was observed in both models during the entire time course of the disease. Smooth muscle cells and intracellular granules in the meningeal vessel wall and epithelial cells in the choroid plexus were clearly VAP-1-positive (Fig. 9), in a similar fashion as described for human tonsil (HEV) and as described in human cerebral vessels [6, 26], but not as extensively. As expected, brain parenchyma lacked VAP-1 (Fig. 9).

## Discussion

In this work, we have evaluated the involvement of VAP-1 during the course of CNS inflammatory lesion development by using a highly selective VAP-1 inhibitor LJP1586, antibodies against VAP-1 and ex vivo autoradiography with a VAP-1 binding radioligand, ^68^Ga-DOTA-Siglec-9. Our results revealed that VAP-1 is expressed and functionally active in the inflammatory CNS lesions in rat models mimicking MS.

Acquisition of MR images from rat brain usually requires the use of a dedicated small animal scanner with the magnetic field strength of at least 7-T, which is capable of producing high-resolution images with high sensitivity [18, 19]. However, our results corroborate that 3-T MRI designed for clinical purposes can be combined with a rat-dedicated brain coil to detect changes in T1 and T2 signal intensities caused by inflammatory CNS lesions. The MRI exhibits normal-appearing signal intensity for pre-active *f*DTH-EAE lesions in T2-weighted and T1-weighted images, and acute *f*DTH-EAE lesions show weak Gd-enhancement in the lesion and hyper-intense lesions in T2-weighted images [19]. The IgG leakage results supported MRI findings, except in acute *f*DTH-EAE lesions with anti-IgG-positivity, highlighting some limitations of 3-T MRI. For *f*MOG-EAE lesions, typical characteristics, such as partially resolved inflammation after acute development, were observed, as shown by a loss in Gd-contrast enhancement and reduced area of T2-weighted hyper-intensity. Immunohistological staining showed marked reduction in IgG leakage for *f*MOG-EAE rats after acute inflammation. The IgG leakage differences between *f*DTH-EAE and *f*MOG-EAE suggest that initially large CNS inflammatory lesions with BBB disruption can be studied by clinical 3-T MRI. The inflammation during the acute phase in MOG-type lesions, however, was surprisingly severe when compared to previous findings. While the *f*MOG-EAE rat model was carefully established as described by Serres and co-workers [18], the mechanisms behind the severe and widespread acute inflammation remain unclear. The effect of LJP1586 treatment, in addition to anti-adhesive effect, decreased the Gd-contrast enhancement in acute inflammatory phase, however leaving the area of IgG leakage unchanged in *f*MOG-EAE rats. Hence, the ability of LJP1586 therapy to contribute to more rapid BBB restoration after its initial breakdown or even prevent the initial BBB breakdown remains questioned. Our results confirm that preclinical research of CNS inflammation does not necessarily require a high magnet MRI to acquire data from large lesions in the rat brain, but small lesions still require high-resolution MRI.

For the first time, we have shown the involvement of VAP-1 in CNS inflammatory lesions in focal EAE. Expression of VAP-1 in normal human brain is well characterized in earlier studies [7, 9, 27]. VAP-1 is occasionally expressed in intracellular granules in brain venules, whereas all neuronal cells are negative for VAP-1 in the brain. In non-clinical studies, the inhibition of semicarbazide-sensitive amine oxidase (SSAO) activity displayed by VAP-1 reduces EAE symptoms in mice during the entire time course of the disease by interfering with the different forms of VAP-1 once the mice have entered the relapse phase [11], but the expression patterns of the membrane-bound form of VAP-1 in EAE has remained undiscovered until now. We have shown, for the first time that the luminal form of VAP-1 is associated with the development of lesions in both the type I and antibody-mediated type II models of MS in rat (Figs. 3 and 4). Yet most importantly, we found that inhibition of VAP-1 activity with small molecular inhibitor LJP1586 decreased the lymphocyte infiltration into brain lesion during the acute inflammation in *f*MOG-EAE rats. At remission, however, the lymphocyte density increased to the same level as without the treatment (Fig. 7b). This could be explained by upregulation of ICAM-1 expression observed in immunofluorescence, which probably compensated the loss of VAP-1-dependent lymphocyte trafficking to inflammation site [28, 29]. Theoretically, this compensation might not be possible in disseminated models of EAE with extensive inflammation, because several adhesion molecules are already heavily involved in the inflammation process and are unable to be further upregulated. Although LJP1586 treatment reduced the lymphocyte recruitment, it was ineffective in reducing the recruitment of OX-42-positive cells and did not translate into diminished demyelination or axonal damage. Therefore, the drug might not prevent the extravasation of key cellular components involved in demyelination, e.g. inflammatory monocytes. LJP1586 treatment might also insufficiently prevent relapses in MS.

Our autoradiography results indicate that VAP-1 targeting radiotracer ^68^Ga-DOTA-Siglec-9 is capable of detecting neuro-inflammatory lesions that exhibit the characteristics of traditional lesions with ongoing demyelination and leukocyte infiltrates. In addition, our data suggest that luminal VAP-1 is not present during the early ‘pre-active’ lesion development in type I MS-like lesions, which was a somewhat unexpected finding. However, during the acute inflammation accompanied by BBB disruption, VAP-1 appears to be translocated to the luminal side of brain vessels at the stages when perivascular leukocyte trafficking is known to take place in *f*DTH-EAE [18]. This might confirm the current assumption that luminal VAP-1 is only recruited at sites of inflammation [2, 12] further suggesting that the initial microglial activation in pre-active lesions is not necessarily associated with leukocyte extravasation from the periphery into the CNS. Thus, luminal VAP-1 is very likely to be expressed in lesion vasculature only after the early small-scale damage in CNS has progressed to pathological changes typical of the developed inflammatory lesions that require amplification of immune response. Therefore, VAP-1 targeted in vivo imaging of new pre-active lesions may not be considered as a promising approach for the early evaluation of pathology in MS.

Perivascular leukocyte infiltration and BBB disruption are known to occur during the relapse in MS [13, 14, 27]. Based on this knowledge, our findings may be translatable to MS during relapses, because luminal VAP-1 is largely expressed in lesion vasculature in type II MS during acute inflammation and overt BBB breakdown. The immunofluorescence staining data (Fig. 4c, g) supports the view that luminal VAP-1 is involved in the disease pathology after the first active relapse and during the widespread neuro-inflammation. Ex vivo autoradiography for *f*MOG-EAE at day 3 revealed this finding, but simultaneously revealed surprising outcomes as ^68^Ga-DOTA-Siglec-9 binding increased for LJP1586 treated rats. As LJP1586 inhibits enzymatic activity of VAP-1/SSAO and not luminal translocation of VAP-1, it might be possible that LJP1586 has minimal interference with binding of radioligand ^68^Ga-DOTA-Siglec-9 to luminal VAP-1 and might even increase ^68^Ga-DOTA-Siglec-9 binding during early treatment [30], although lymphocyte trafficking is found to be reduced in acute inflammation. Interestingly, the CNS lesion area expanded in LJP1586-treated rats. Unlike *f*DTH-EAE, ^68^Ga-DOTA-Siglec-9 binding for untreated *f*MOG-EAE remained constant until day 14, which supports the finding that luminal VAP-1 is expressed on restored BBB in *f*MOG-EAE. In addition, the endothelium especially in MOG-type lesions seems to undergo a structural transformation from regular flat-walled endothelium to HEV-like endothelium similar to that observed, for example, in skin inflammation [6, 30]. In contrast to MOG-type lesions, the endothelial cells in DTH-type lesions mostly displayed flat-walled morphology.

The myelin status of *f*MOG-EAE has previously been characterized [31] and our observations are in the line with those. The *f*MOG-EAE rats showed extensive demyelination throughout the grey matter of injected brain hemisphere and underwent remyelination during the follow-up period of 14 days as observed in LFB stained sections. Axonal damage and neuronal cell death have been demonstrated in EAE lesions [31, 32]. In this study, the maximal axonal damage was elucidated at day 3 in *f*MOG-EAE according to NFP staining.

We observed microglial activation in pre-active DTH-type lesions prior to the development of active inflammatory lesion visible in MRI. Inflammatory cells cannot be detected 4 weeks after intracranial injection of BCG [22], so the detected microglial activation most likely results from the peripheral activation of CFA supplemented with BCG. This finding may be comparable to findings in human MS [15]. In addition, microglial activation in EAE is shown to be more widespread than has earlier been understood [3] and it is known to occur also in areas of NAWM [31]. The present study shows that NAWM lacks VAP-1 as the luminal VAP-1 is upregulated only in the hypercellular core of lesions. As OX-42 staining recognizes monocytes, macrophages, granulocytes, dendritic cells and microglia [33], the identified correlation between OX-42 positivity and VAP-1 targeted autoradiography may result from VAP-1-mediated lymphocyte trafficking to lesion areas. The observed patterns of the luminal VAP-1 in EAE demonstrate that its role in MS might be related to provoking inflammation in demyelinated lesions while the function of VAP-1 in driving new lesion formation may be less significant.

Both investigated stereotactically injected focal models exhibit some limitations compared to more traditionally used disseminated EAE [34–36] such as they lack the ability to create new lesions during the disease course. In addition, the immunopathology of focal EAE lesions strongly depends on the site of lesion initiation [31, 37]. To minimize this problem, we primed Lewis rats with MOG_35-55_ encephalitogenic peptide fragment suitable for studying the onset and development of EAE and testing efficacy of potential therapeutics, whereas MOG_1-125_ is recommended for EAE immunization for testing B-cell targeting therapeutics [36].

Luminal VAP-1 expression has shown to play a pivotal role in regulating leukocyte recruitment in acute and chronic inflammations in mice [38], but the function of VAP-1 in a chronic focal model of EAE mimicking progressive MS still needs further investigation. Because the luminal VAP-1 levels in the endothelial cell surface of lesion vasculature are elevated after the acute inflammation phase, the levels of luminal VAP-1 may affect the clinical status of EAE [11, 38].

## Conclusions

Based on the findings of the present study, clinical 3-T MRI can offer a feasible method for visualization of inflammatory lesions in rats. Moreover, the membrane bound form of VAP-1 is actively associated with the lymphocyte transmigration during acute inflammation of MS-like lesions in rats. The VAP-1 expression remains on the BBB even after the acute lesion has subsided and is thus potentially providing a molecular support for leukocyte trafficking to the lesion site.

## Abbreviations

%ID: Percentage of injected radioactivity dose
^68^Ga-DOTA-Siglec-9: Gallium-68-labelled 1,4,7,10-tetraazacyclododecane-*N,N′,N′′,N′′′*-tetraacetic acid conjugated sialic acid-binding immunoglobulin-like lectin 9 motif containing peptide
BBB: Blood-brain barrier
BCG: Bacillus Calmette-Guérin
CFA: Complete Freund’s adjuvant
CNS: Central nervous system
EAE: Experimental allergic encephalomyelitis
*f*DTH-EAE: Focal delayed-type hypersensitivity model of experimental allergic encephalomyelitis
FITC: Fluorescein isothiocyanate
*f*MOG-EAE: Focal myelin oligodendrocyte glycoprotein induced model of experimental allergic encephalomyelitis
FOV: Field of view
Gd: Gadolinium
H&E: Hematoxylin-eosin
HEV: High endothelial venule
i.v.: Intravenous(ly)
ICAM-1: Intercellular adhesion molecule-1
IFA: Incomplete Freund’s adjuvant
IgG: Immunoglobulin G
INF-γ: Interferon-γ
LFB: Luxol fast blue
LJP1586: Z-3-Fluoro-2-(4-methoxybenzyl) allylamine hydrochloride
MRI: Magnetic resonance imaging
MS: Multiple sclerosis
NAWM: Normal appearing white matter
NFP: Neurofilament protein
PBS: Phosphate-buffer saline
PET: Positron emission tomography
PLP: Periodate-lysine-paraformaldehyde
PSL/mm^2^: Photostimulated luminescence per square millimeter
ROI: Region of interest
RR-MS: Relapsing-remitting multiple sclerosis
s.c.: Subcutaneous(ly)
SSAO: Semicarbazide sensitive amine oxidase
TB: Mycobacterium tuberculosis
TE: Echo time
TNF-α: Tumor necrosis factor-α
TR: Time of repetition
TSE: Turbo spin echo
VAP-1: Vascular adhesion protein-1
